# Cerebral Surgery

**Published:** 1895-08-03

**Authors:** 


### PROGRESS IN SURGERY.

### CEREBRAL SURGERY.

(*Continued from page* 290.)

**Cerebral Abscess.**—Professor Murri, of Bologna, in a series of lectures delivered before the Lombard Medical Association,[15] considers the difficulties of diagnosis in cases of cerebral abscess of the chronic form. So great does he believe this difficulty to be in the early age, and so fatal is the disease if left to a late stage, that Professor Murri advocates that early exploration should be undertaken much more frequently than is now done. He thinks that we must not wait for exact diagnosis, but when we have reasonable grounds for suspecting abscess it is right to explore, as many cases now die undiagnosed. Many very instructive cases are reported by him, showing the great difficulty there may be in diagnosis, and he points out that, though middle ear disease, and injury to the head, or caries of the skull are all common causes of abscess, yet we must not forget that with such conditions tumour is quite common, and it is between tumour and abscess that often the greatest difficulty in differential diagnosis arises. He agrees with

Horsley and others as to frequency of normal or subnormal temperature in chronic abscess. Dr. F. Deansley[16] has collected records of twenty-one cases of cerebellar abscess from the periodicals of the last ten years. He finds that purulent otitis media was present in all, and that the abscess lay in the anterior part of the lateral lobe of the cerebellum on the same side as the disease of the ear, in all but two cases, and in one of these it was situated on the inferior surface of the middle lobe and in the other in the flocculus. The abscess was single in every case,, and in the great majority had arisen by direct local extension of infective inflammation from the posterior surface of the petrous bone. Twelve of the cases presented serious complications in addition to the disease of the middle ear, such as extradural abscess, sinus phlebitis, localised or general meningitis, or a second abscess in the cerebrum. Sinus phlebitis with or without thrombosis occurred in seven, and extradural abscess in six. Headache was present in all, but the position varied greatly. The temperature was only raised throughout in one case. Respiration was in many cases irregular, and in two cases of Cheyne-Stokes rhythm. In nearly every case in which the mental condition was noted there was well-marked slowness of cerebration and general apathy. In seven cases there was some paralysis or anæsthesia, excluding lesions of the facial nerve from the ear disease. In one there was paresis and rigidity of the arm on the same side as the abscess from pressure on the motor track of the same side below the decussation of the pyramids. In the other cases the cranial nerves were affected. The author considers that paralysis of cranial nerves when brain abscess has been diagnosed from other evidence, is in favour of its being situated in the cerebellum rather than in the tempero-sphenoidal lobe, since it is usually due to pressure or local meningitis, which he thinks more likely to extend to the nerves from the cerebellum than the more distant tempero-sphenoidal lobe. Rigidity of the neck and retraction of the head was present in one-third of the cases, and conjugate deviation of the head and eyes towards the side opposite the cerebellar lesion was present in two cases; nystagmus was only present in four cases. Affections of equilibrium were present in seven cases, always in the form of vertigo, and often of staggering gait or a tendency to fall, always to the same side, without any paralysis to account for it. In discussing the differential diagnosis of the complications of middle ear disease, Dr. Deansley says that he thinks neither mental sluggishness nor a persistent subnormal temperature occurs unless there is mischief inside the dura mater; and that if either of these symptoms are associated with slow pulse and respiration, vomiting or optic neuritis, either abscess or meningitis is certainly present. As abscess is so rarely multiple, any evidence pointing to implication of distant parts of the brain or distant cranial nerves, he thinks a strong indication of general meningitis. Twelve of the twenty-one cases were operated on, and six recovered. One of the successful cases is reported by the author. The patient was fourteen years of age, and symptoms pointed to cerebral abscess, secondary to middle ear disease, but did not show clearly whether the abscess was in the cerebrum or cerebellum. Hence the brain was explored by Dean's method.[17] A trephine disc was removed over the lateral sinus, and then the bone cut away first upwards so as to explore the tempero-sphenoidal lobe, and then downwards beneath the tentorium so as to explore the lateral lobe of the cerebellum. Too small a trochar was employed first, and the pus missed, then on introducing a larger sized one in the same place it flowed out. Another case in which the brain was explored for abscess by Dean's method is recorded by Mr. Morton.[18] In this case (as in Dean's case) the lateral sinus, tempero-sphenoidal lobe, and the cerebellum were explored by cutting away the bone upwards and downwards. Mr. Murray reports[19] three successful cases of cerebral abscess in which the abscess was drained. In one case the abscess followed an acute otorrhœa; in another—a cerebellar abscess—sighing respiration was present. Dr. Drummond[20] records a very remarkable case of cerebellar abscess, in which though the abscess was situated in the right lateral lobe, and no lesion of any other part of the brain could be discovered, yet there was paralysis and convulsions in the right leg, arm, and face. Direct pressure on the motor tract below the decussation of the pyramids would not explain the convulsions or the implication of the face in the paralysis.

[15] Lancet, Jan. 5 h, 12th, 26th, Feb. 2nd. [16] Ibid, Dec. 8, 1894, p. 1,339. [17] Lancet, July 30th, 1892. [18] St. Bart. Hosp. Reports, 1895. [19] Brit. Med. Journ., Jan. 5th, 1895. [20] Lancet, July 28th, 1894.

